# Design and Experimental of the Soil Removal Device for Root-Soil Complex of *Gentian* Imitating the Percussion of Woodpeckers

**DOI:** 10.3390/biomimetics9080479

**Published:** 2024-08-08

**Authors:** Hongguang Cui, Li Du, Zhanqiu Xie, Wei Zhong, Dehui Xu, Weiming Bian, Long Jiang, Tiejun Wang, Liyan Wu

**Affiliations:** 1 College of Engineering, Shenyang Agricultural University, Shenyang 110866, China; chg7763@syau.edu.cn (H.C.); duli10345@163.com (L.D.); 15941257236@163.com (Z.X.); zw763252209@163.com (W.Z.); huihuihuia2001@163.com (D.X.); tiejunwang@syau.edu.cn (T.W.); 2 Fushun Agricultural and Rural Development Service Center, Fushun 113000, China; bianweiming@126.com; 3 Fushun Agricultural Comprehensive Administrative Law Enforcement Team, Fushun 113006, China; jianglong4409305@163.com

**Keywords:** *Gentian* root and soil separation, bionic design, woodpecker, percussion soil removal, cam mechanism

## Abstract

A soil removal device for the root-soil complex of *Gentian* imitating the percussion function of a woodpecker was designed to improve the soil removal efficiency of harvesting devices for rhizome-type traditional Chinese herbal medicines. Based on the physical parameters of roots and the root-soil complex of *Gentian*, the structure parameters of the striking arm and the actual profile of the cam are determined according to the physical parameters when the woodpecker knocks on the tree. The key parameters that affect the working performance of the soil removal device and their suitable value ranges have been identified through the impact test and analysis of the root-soil complex of *Gentian*. The mass of the striking hammer, the swing angle of the striking arm, and the rotation speed of the cam were taken as the experimental factors and the soil removal rate and the energy consumption per hammer percussion were taken as the experimental indicators. The ternary quadratic orthogonal regression combination experiment was carried out using Design-Expert. The regression model of the influence factors and evaluation indicators was established through the analysis of variance. The interaction effects of the influence factors on the indicators were analyzed using the response surface method. Using multiobjective optimization method, the optimal parameter combination was obtained as that of the mass of the striking hammer of 0.9 kg, the swing angle of the striking arm of 47°, and the rotation speed of the cam of 100 r/min, then the soil removal rate was the maximum and the energy consumption of single-hammer knocking was the minimum, with the values of 89.12% and 31.21 J, respectively. This study can provide a reference for the design and optimization of soil removal devices for rhizome-type traditional Chinese herbal medicines.

## 1. Introduction

Roots are important medicinal parts of Chinese herbal medicines, which are generally harvested in the form of root-soil complexes, and root-soil separation is an important part of harvesting. Improving the desoiling effect of the root-soil complex could improve the economic efficiency of farmers, reduce the time required for subsequent cleaning and drying processes, and reduce the loss of effective ingredients. Therefore, it is of great significance to develop a soil removal device for rhizome-type traditional Chinese herbal medicines.

*Gentian* (*Gentiana scabra* Bunge.) is a typical rhizome-type traditional Chinese herbal medicine that belongs to the *Platycodonaceae* family of perennial herbs. It is mainly produced in regions such as Northeast China and Yunnan and its rhizome is used medicinally to purge excessive heat from the liver and gallbladder, among other benefits [1]. Due to the small plant spacing of *Gentian*, when it is harvested, the roots of multiple plants are intertwined and it is difficult to remove the soil. The mechanism for separating the *Gentian* roots from the soil remains to be clarified.

Currently, the separation of *Gentian* root-soil mainly relies on manual labor. A single person could separate 5.0–8.0 kg of *Gentian* per hour from the root-soil complex of *Gentian*. Through manual operation, it has been found that by hammering the bud position of the root-soil complex of *Gentian*, the aggregated soil in that area can be loosened and, combined with shaking, the intertwined roots of multiple plants could be separated and a single *Gentian* root without soil can be obtained.

Since the 1990s, China has begun to conduct research on root-soil separation machinery for rhizomatous Chinese herbal medicines, such as a root-soil separation device for deep-rooted herbal medicines developed by the South China Agricultural University, which put forward a set of modes of “double vibration damping, double-stage flexible conveying, and double-type root-soil separation”. Chen et al. designed a double-roller root-soil separation device for the desoiling of the root system of the Chinese traditional medicine *Tiger Balm* [2]. The Zhang team of the Kunming University of Science and Technology researched a harvesting and separation device for *Panax pseudoginseng*, including a chain-bar conveyor separation device designed by Yu et al. to realize the *Panax pseudoginseng* root-soil separation requirements [3]; Cui et al. designed a two-stage vibrating screen, which resulted in the improvement of the sieve clean rate [4]. Wang et al. carried out an analysis of the operation mechanism and parameter optimization on the conveying and separating device of the *Panax pseudoginseng* harvesting machine [5]. Xue et al. designed a *Panax pseudoginseng* conveying and separating device: the shaking up and down of the vibrating screen made the root and soil more fully separated and the lifting rod structure with finger rubber and two-stage lifting transmission mode was chosen for the separation and cleaning of *Panax pseudoginseng* root and soil [6]. Song et al. developed the B-1200 *Pingbeimu* herb harvester, which adopted the vibrating excavation and soil removal structure, and found that the loss rate and damage rate of *Pingbeimu* are less than 5% in the experiment [7]. The Chinese herbal medicines mentioned above belong to the single-root herbs, which have achieved better experimental results using vibration harvesting in excavation. However, for *Gentiana*, with multiple intertwined roots, the vibration for root-soil separation is not thorough enough. Instead, high-energy percussion is required.

Currently, biomimetic design is applied in agricultural machinery. Du et al. mimicked the arrangement of picking fingers to design a pepper-picking device, which reduces the ground drop loss during the pepper-picking process and improves the net recovery rate [8]. Yuan et al. designed a high-traction track grouser based on the structure of an ostrich’s foot sole, significantly improving the traction of the track in wet and soft rice fields [9]. Lu et al. designed an optimized protuberance-type bionic pressing wheel taking the dung beetle’s protruding head structure, which had better soil antiadhesive performance [10]. Chen et al. designed a bionic walking wheel based on the structural morphology and movement mode of the feet of waders living in marshes and mudflats, which had better antisinking performance [11]. Ding et al. proposed a bionic knocking (i.e., imitating the way of manually processing pecans) shell-breaking method and developed a bionic knocking pecan shell-breaking machine to achieve the operational requirements of high shell-breaking and low kernel damage rate [12]. Zhao conducted research on the bionic pecking mechanism by exploring the pecking motion mechanism of woodpeckers for pecking sampling technology and the bionic design of an impact hammer [13]. Many scholars in China have conducted related studies on woodpecker [14,15,16] and material tapping [17,18,19,20] respectively.

In this paper, a bionic soil removal device for the root-soil complex of *Gentian* imitating the percussion of woodpeckers is developed. By imitating the movement process and other comprehensive principles of woodpeckers pecking the trunk, it determines the structural dimensions of the key parts of the soil removal device. Through the prototype experiments, the knocking mechanism parameters were optimized to improve the effect of the *Gentian* herb root-soil complex root-soil separation. It provides a basis for the study of the root-soil separation of the herb root series of Chinese herbal medicines harvesting machinery.

## 2. Materials and Methods

### 2.1. Characteristics Parameters of the Root-Soil Complex of Gentian

**Figure 1:**
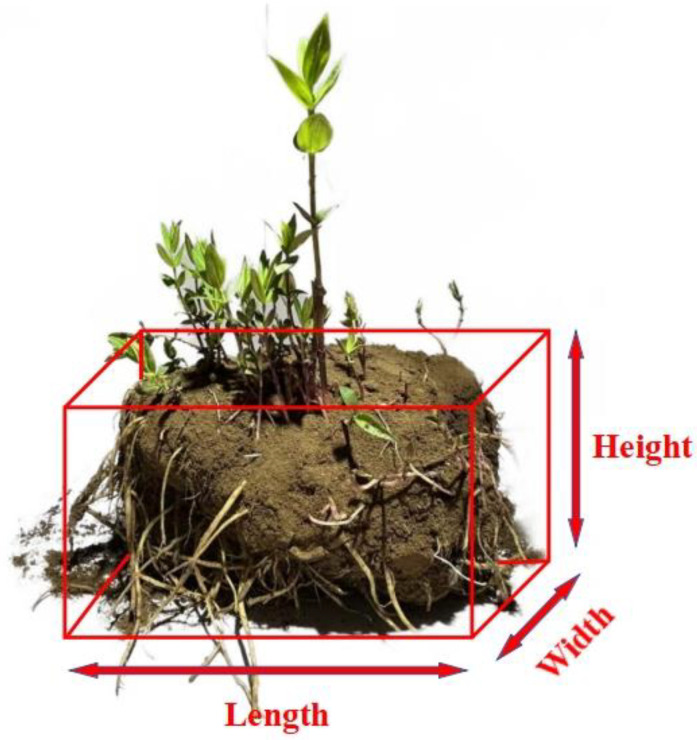
Schematic diagram of the measured geometric parameters of the root-soil complex of *Gentian*.

The root-soil complexes of *Gentian* from Qingyuan, Liaoning, were selected as the experimental materials. The five-point method was used for field sampling and a total of 50 root-soil complexes of *Gentiana* were selected. As shown in Figure 1, the geometric parameters were measured. Length and width were two physical quantities measured perpendicular to the sprout’s growth direction of a root-soil complex of *Gentian* and height was measured along the sprout’s growth direction. The range in length is 93–121 mm, in width is 84–113 mm, and in height is 69–96 mm. The soil moisture contents were measured using a Jingheng halogen rapid moisture meter (Yixinde Instrumentation Co., Ltd., Taizhou, China). The ranges in the soil moisture contents are 17.6–21.4%. The physical properties of the materials provide necessary theoretical support for the mechanical structure and material selection of the device.

### 2.2. Machine Structure and Technical Parameters

#### 2.2.1. Machine Structure

**Figure 2:**
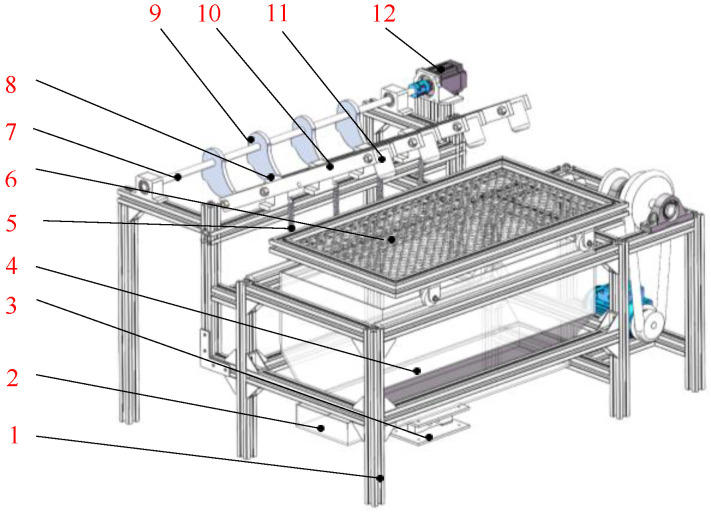
The soil removal device for the root-soil complex of *Gentian* imitating the percussion of woodpeckers. 1—frame; 2—soil-weighing tray; 3—soil-weighing detection system; 4—retaining box; 5—tension spring; 6—Gentian-soil separation sieve; 7—camshaft; 8—support base; 9—cam; 10—striking arm; 11—striking hammer; 12—motor.

The soil removal device for the root-soil complex of *Gentian*, which imitates the percussion action of a woodpecker, mainly consists of a woodpecker-imitating percussion mechanism, transmission system, separating sieve, fallen soil quality detection system, and frame. The structure is shown in Figure 2. Under the percussion actions of the woodpecker-imitating pecking mechanism, the root-soil complex of *Gentian* can be broken up and the soil detached through repeated percussion actions.

#### 2.2.2. Working Principle and Technical Parameters

The percussion mechanism of the soil removal device for the root-soil complex of *Gentian*, imitating the percussion action of a woodpecker, primarily consists of a camshaft, cam, support brackets, bionic striking arm, striking hammer, and tension springs. The camshaft was fixed to the frame through bearing pedestals and the cams were fixed side by side on the camshaft at staggered angles, located directly behind the bionic striking arms. The bionic striking arms were fixed to the frame through support brackets and could rotate around the support brackets. Every bionic striking arm was equipped with a lever roller at the rear end and a fixed striking hammer at the front end. When the percussion mechanism was working, the lever roller was pressed by the cam and rolled along the contour curve of the cam. During the ascending stroke of the cam, the striking hammer was lifted by the cam’s pressure. When the lever roller separated from the cam, the striking hammer, under the combined forces of its own weight and the spring tension on the striking arm, struck the root-soil complex of *Gentian*. Under the impact force action, the aggregated soils were broken apart and the intertwined roots of the *Gentian* were loosened, ultimately achieving their separation. After the percussion action, the broken soil fell through the separating sieve into the soil-weighing tray while the *Gentian* roots remained on the upper layer of the separating sieve; thus, the separation of the roots and soil was completed.

**Figure 3:**
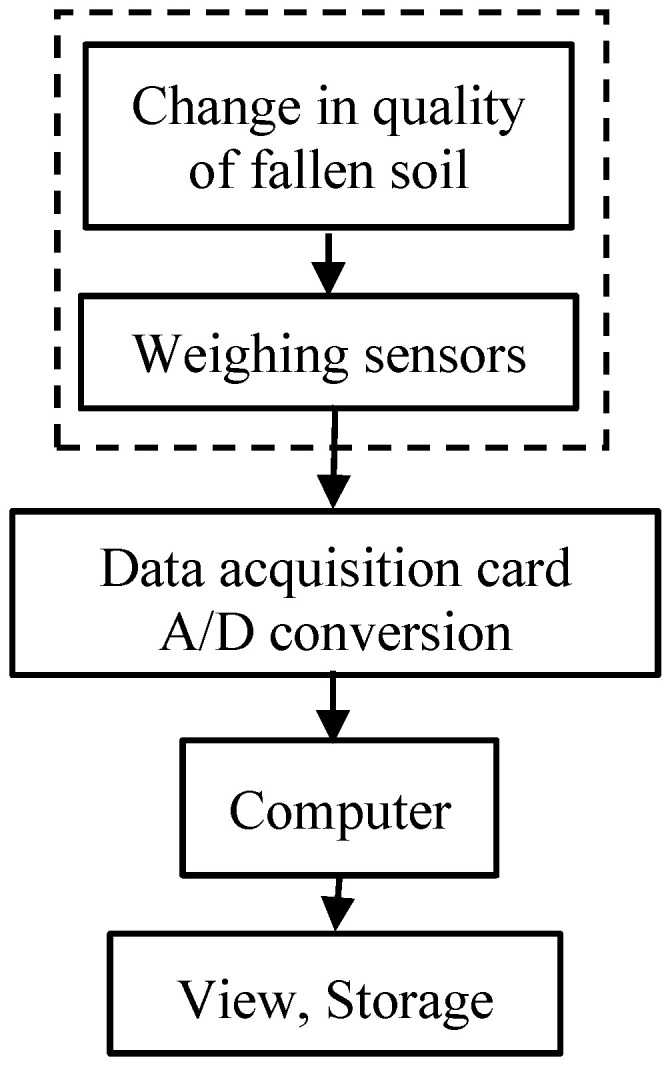
Schematic diagram of the soil removal weighing detection system for the root-soil complex of *Gentian*.

The soil removal weighing detection system for the root-soil complex of *Gentian* is shown in Figure 3. It was mainly composed of a soil-receiving tray, a weighing sensor, a transmitter, a data acquisition card, and a computer. It was developed on LabVIEW 2021 Chinese trial version software to realize the display of detection data and storage of detection.

When the root-soil complex of *Gentian* was knocked, the detached soil fell through the sieve holes into the soil-receiving tray. The weight of the soil was detected by the weighing sensor and transmitter (models DYX-306 and DY-510, Bengbu Day ang Sensor System Engineering Co., Ltd., Bengbu, China). The data were collected by the data acquisition card (NI USB-6009), the A/D conversion, and were transmitted to the computer. The virtual instrument interface displayed the curves and data.

The main technical parameters of the soil removal device for the root-soil complex of *Gentian* imitating the percussion of woodpeckers are shown in Table 1.

### 2.3. Design of the Soil Removal Device for the Root-Soil Complex of Gentian Imitating the Percussion of Woodpeckers

#### 2.3.1. Bionic Principle of Bionic Woodpecker Percussion Mechanism

Woodpeckers obtain food or build nests through their percussion motions on trees. The percussion behavior of woodpeckers is characterized by its speed and high frequency but effectively prevents brain damage from impact. The uniqueness of woodpeckers’ percussion behavior has important engineering implications in structural design and mechanical principles.

Scholars have conducted quantitative research on woodpeckers’ percussion motion. Stark et al. found that the percussion frequency of woodpeckers can reach 10–22 times per second [21]. Vincent et al. proposed that woodpeckers can achieve a percussion speed of 3.6 m/s at a distance of 70 mm [22]. May et al. used high-speed cameras to capture the head movement of woodpeckers during percussion and found that the head of woodpeckers in the percussion process is approximately straight and the resulting impact acceleration is 1.0 kg [23].

Yoon et al. found in their observation of the percussion motion of woodpeckers that the beak of woodpeckers usually strikes the tree trunk vertically while the body rotates around the tail vertebrae [24]. The research of Collins et al. further showed that the continuous motion of woodpeckers can be simulated as a resonant system with a periodic forcing function [25].

Inspired by these research results, this study designed a percussion mechanism with a periodic reciprocating motion similar to the percussion motion of woodpeckers. The mechanism aims to strike the root-soil complex of *Gentian* with the necessary energy and speed to achieve effective separation of the root and soil.

#### 2.3.2. Simplified Modeling of Woodpecker Percussion Motion

**Figure 4:**
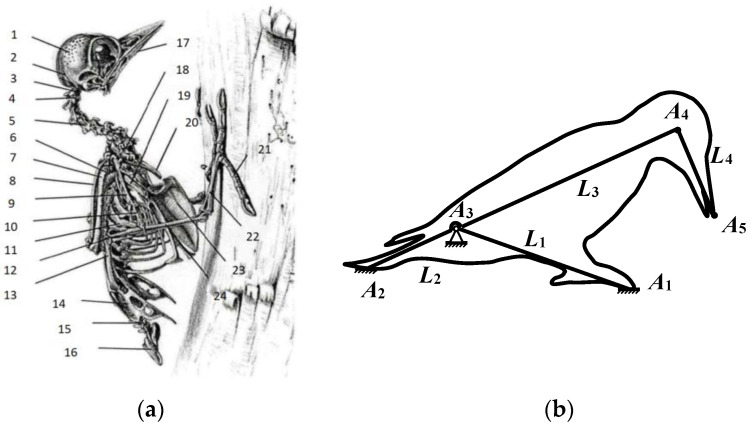
Simplified model of woodpecker skeleton and pecking motion. (**a**) skeletal structure diagram, 1—skull, 2—hyoid bone, 3—cricoid vertebrae, 4—pivot vertebrae, 5—cervical vertebrae, 6—humerus, 7—ulna, 8—radius, 9—metacarpals, 10—phalanges, 11—ribs, 12—knee bone, 13—femur, 14—ilium, 15—caudal vertebrae, 16—tailbone, 17—mandible, 18—thoracic vertebrae, 19—coracoid, 20—clavicle, 21—toe bone, 22—tarsal metatarsal bone, 23—keel bone, 24—tibia; (**b**) is a simplified pecker movement model.

Given the intricate skeletal structure and unique force exertion mechanism of woodpeckers, it was discovered through analysis that the pecking behavior can be emulated by a simplified kinematic pair. Based on the morphological characteristics of woodpeckers and the movement patterns of their crucial points, a simplified model of woodpecker percussion motion was constructed with reference to the woodpecker’s full-body skeletal structure diagram (Figure 4a [13]), as illustrated in Figure 4b). This model provides theoretical support for subsequent mechanical analysis and bionic design.

When constructing a simplified model of the woodpecker’s percussion motion, considering that the upper and lower torso parts hardly deform during the motion, they can be simplified as rigid rods. The complex rotation of the woodpecker’s knee bone is simplified to a ball joint. In the model as shown in Figure 4b, *A*_1_ is the woodpecker’s claw, which plays the role of fixed support. *A*_2_ is the tail, which is simplified from the ilium, coccyx, and tailbone, providing a stable balance for the entire structure. *A*_3_ is the knee bone, which is the key rotation point for the woodpecker to perform percussion motion. *A*_4_ is the skull and *A*_5_ is the beak, serving as the main action point for percussion motion. *L*_1_ represents the tibia and toe bone of the woodpecker, which are simplified to form an integrated leg support rigid rod. *L*_2_ is a rigid rod simplified from the knee bone to the tailbone, while *L*_3_ is a rigid rod simplified from the skull to the knee bone. Since the woodpecker is driven by its own internal strength but in mechanical structures, external forces are required to drive it, *L*_2_ and *L*_3_ can be further approximated as a rigid rod with *A*_3_ as a fixed hinge point forming a lever structure. *L*_4_ is the rigid rod that connects the beak to the skull. By applying a driving force at the tail *A*_2_, the percussion motion of the woodpecker can be simulated. The simplified percussion motion process can be described as follows: when the tail *A*_2_ is subjected to external forces, the torso part composed of *L*_2_ and *L*_3_ is rotated around *A*_3_, which causes the end *A*_5_ perpendicular to *L*_3_ to generate large speed and energy, thus completing the percussion motion.

**Figure 5:**
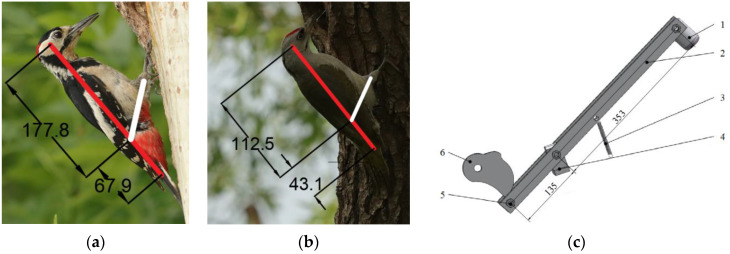
Simplified model of the proportion of the picture of the woodpecker pecking. (**a**,**b**) are measuring parameters on the pictures of woodpeckers pecking; (**c**) the imitating woodpecker percussion mechanism, 1—striking hammer; 2—striking arm; 3—tension springs; 4—support brackets; 5—pressing roller; 6—cam.

In order to determine the size parameters of each mechanism in the simplified model, this study selected 50 pictures of woodpeckers pecking and obtained the ratio of the distance (from the skull to the knee bone) to the length (from the knee bone to the tailbone) by measuring the parameters of the body structure of the woodpecker in these pictures. As shown in Figure 5a,b, the specific range of this ratio was obtained through calculation, which corresponds to *L*_3_:*L*_2_ = 2.51 to 2.64:1 in Figure 4b.

#### 2.3.3. Structure Design of the Bionic Woodpecker Percussion Mechanism

The imitating woodpecker percussion mechanism adopts a “lever-spring-cam” mechanism to simulate the joint structure and force generation method of a woodpecker, as shown in Figure 5c. The mechanism consists of core components such as a striking hammer, striking arm, tension spring, support brackets, pressure roller, and cam, which enables the mechanical system to imitate the striking motion of a woodpecker.

The function of the cam is to provide an external driving force for the mechanism, acting on the pressure roller to cause the lever mechanism to undergo regular reciprocal motion. The function of the lever is to convert a small but constant frequency of power into a larger speed and energy for the striking hammer, thereby achieving loosening and separation of the root-soil.

The rear end of the bionic striking arm can rotate on the support brackets *A*_3_ to complete the lifting and falling actions of the striking hammer *A*_5_. The tail end *A*_2_ of the striking arm is equipped with a rotatable pressure roller, which effectively reduces the rigid friction between the cam and the pressure roller, ensuring the stable operation of the device.

The striking hammer *A*_5_ is mounted on the top end *A*_4_ of the striking arm. The mass of the striking hammer and the effective length of the striking arm determine the amount of the crushing energy of soil during the operation process. The front end of the striking arm is fixed on the frame by installing a tension spring. When the cam acts on the pressure roller, the spring accumulates elastic potential energy through mechanical stretching during the lifting process of the striking arm. When the cam withdraws the force on the pressure roller, that is, the striking arm acts together with the self-weight of the striking hammer under the action of spring tension, it has a certain amount of the crushing energy of soil during the falling process of the striking arm. By adjusting the pretightening force of the spring, the amount of the crushing energy of soil can be adjusted to a certain extent.

#### 2.3.4. The Parameter Calculation of the Bionic Woodpecker Percussion Mechanism

**Figure 6:**
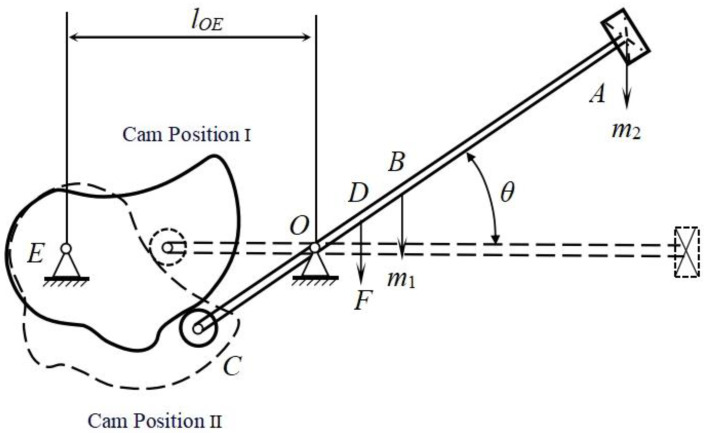
Mechanical analysis diagram of the bionic woodpecker percussion mechanism.

The mechanical analysis of the bionic woodpecker percussion mechanism is shown in Figure 6. Taking the horizontal plane as the zero-potential energy plane, the pressure rod roller C installed at the tail end of the striking arm is pressed down by the cam at solid line position I. This downward movement causes the striking arm *AC* to rotate around the O-axis, lifting the striking hammer *A* upwards. When rotating an angle *θ*, the cam moves to the dash line position II and the pressure at point *C* disappears. Under the effect of its own gravity and the spring tension at point *D*, the striking hammer *A* moves downward. The entire system is a conservative system, where gravitational potential energy and elastic potential energy are converted into kinetic energy; thus, the required soil fragmentation energy will be obtained.

The movement of the bionic woodpecker percussion mechanism can be specifically divided into three parts:

(1) Swing and lift phase of the striking arm. Assuming the angular velocity of the striking arm is *ω*, its rotational angular velocity is determined by the motion pattern of the slave part. For the selection of the cosine acceleration motion pattern for the slave part, the angular velocity at the beginning and ending points of the trajectory are both zero, so *ω*_1_ = 0.

(2) Hammer falling. Starting from the end of the far-rest segment and ending at the instant when the striking arm makes contact with the root-soil complex of *Gentian*. By applying the rotation moment theorem for rigid bodies and the kinetic energy theorem for a system of primes, formulae are obtained:

(1) $$\Delta E_{k}=\frac{1}{2}J_{O}(\omega_{2}^{2}-\omega_{1}^{2})$$

(2) $$J_{O}=m_{1}l_{OB}^{2}+\frac{1}{12}m_{1}l_{AC}^{2}+m_{2}l_{OA}^{2}+m_{3}l_{OC}^{2}$$

(3) $$\Delta E_{p}=m_{1}gl_{OB}\sin\theta +m_{2}gl_{OA}\sin\theta -m_{3}gl_{OC}\sin\theta +\frac{1}{2}k\Delta \delta^{2}$$

(4) $$\Delta \delta =l_{OD}\sin\theta$$

(5) $$\Delta E_{k}=\Delta E_{p}$$

where, *m*_1_—the mass of the striking arm, kg; *m*_2_—the mass of the striking hammer, kg; *m*_3_—the mass of the roller, kg; *θ*—the swing angle of the striking arm, (°); *J*_O_—the rotational inertia of the system, (kg·m^2^); ∆*δ*—the deformation of the spring, mm; *ω*_1_—the angular velocity at the maximum swing angle, (r/min); *ω*_2_—the angular velocity at the instant when the striking arm contact with the root-soil complex of *Gentian*, (r/min).

(3) Heavy striking. The process of heavy striking breaking soil is an instantaneous collision where energy conservation is always satisfied during the collision [26,27]. The work performed by the impact force generated during the collision in the direction of the deformation of the root-soil complex of *Gentian* and the energy consumed by the friction of the mechanism are neglected. The energy range for heavy striking breaks soil is set at 1.4 J to 3.1 J. By the kinetic energy theorem for a system of primes, the following formula is obtained:

Δ*EK* = *C* (6)

where, *C*—constant.

After analysis and calculation, the parameters of the bionic woodpecker percussion mechanism are shown in Table 2.

#### 2.3.5. Cam Design of the Bionic Woodpecker Percussion Mechanism

The shape of the cam profile curve depends on the motion law of the slave part and selecting the appropriate motion law of the slave part is the key to designing a reasonable cam profile curve [28]. When using the bionic woodpecker percussion mechanism for soil removal operations of the root-soil complex of *Gentian*, multiple heavy strikes are necessary. Therefore, the cam curve should include a lift segment and a distant rest segment.

According to the actual requirements, the sum of the cam motion rotation angles of the push transition segment, push segment, and distant rest segment is set to 120°. The working states of the soil removal device for the root-soil complex of *Gentian* imitating the percussion of woodpeckers are low speed and medium load, so the cam mechanism designed here is a typical “force-locking” cam mechanism [29]. According to the operation requirements, the initial and final angular velocities during the push process should be zero and the motion should be stable during the push process. Therefore, the cosine acceleration motion law is selected in this paper.

**Figure 7:**
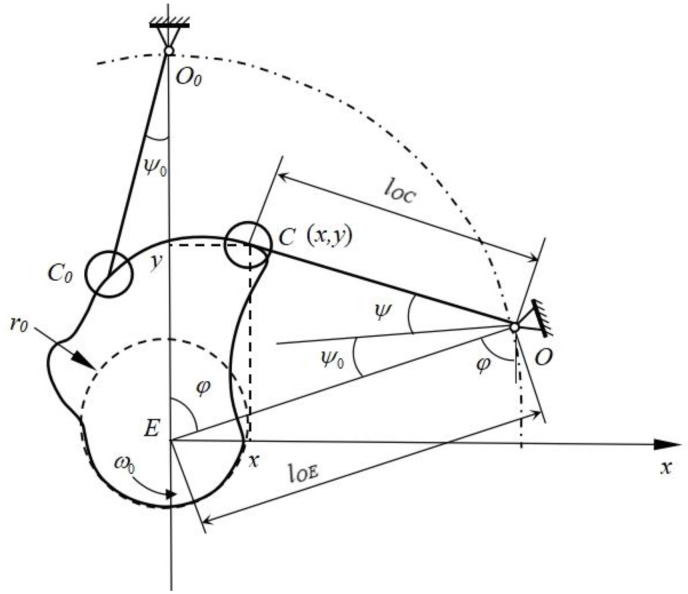
Diagram of cam profile curve.

Based on the radius of the press lever roller *r_g_*, the radius of the cam base circle *r*_0_, the length of the swing lever *l_OC_*, the distance from the swing center to the cam rotation center *l_OE_*, and the motion law of the slave part *Ψ* = *Ψ*(*φ*), a right-hand rectangular coordinate system is established with the cam rotation center as the origin and the line connecting the cam rotation center and the swing center as the positive *y*-axis, as shown in Figure 7. The inversion method is used to determine the coordinates of the center *C* of the swing lever roller.

(7) $$\left\{\begin{array}{l} x=l_{OE}\sin\varphi -l_{OC}\sin\left[\psi (\varphi )+\psi_{0}+\varphi \right]\\ y=l_{OE}\cos\varphi -l_{OC}\cos\left[\psi (\varphi )+\psi_{0}+\varphi \right] \end{array}\right.$$

From the design of the cam profiles using graphical methods, it is known that in a disk cam mechanism with a roller slave part, the actual cam profile is the envelope of the theoretical cam profile with respect to the original family of rollers. Assuming that the coordinates of point *C*′ on the actual cam profile are (*x*′, *y*′), the equation of the actual cam profile can be expressed as:

(8) $$\left\{\begin{array}{l} x^{\prime }=x-r_{g}\cos\beta \\ y^{\prime }=y-r_{g}\sin\beta \end{array}\right.$$

where, *β*—angle between the common normal and the axis, (°).

(9) $$\sin\beta =\frac{\left(dx/d\varphi \right)}{\sqrt{{\left(dx/d\varphi \right)}^{2}+{\left(dy/d\varphi \right)}^{2}}}$$

(10) $$\cos\beta =-\frac{\left(dy/d\varphi \right)}{\sqrt{{\left(dx/d\varphi \right)}^{2}+{\left(dy/d\varphi \right)}^{2}}}$$

(11) $$\frac{dy}{d\varphi }=-l_{OE}\sin\varphi +l_{OC}\sin\left[\psi (\varphi )+\psi_{0}+\varphi \right](\frac{d\psi }{d\varphi }+1)$$

(12) $$\frac{dx}{d\varphi }=l_{OE}\cos\varphi -l_{OC}\cos\left[\psi (\varphi )+\psi_{0}+\varphi \right](\frac{d\psi }{d\varphi }+1)$$

Taking a calculation interval of 5° for the cam rotation angle of each segment, the distance from point *C* to the center of the base circle for each interval is calculated using Equations (13) and (14) and the base circle radius is subtracted to obtain the push of each segment. Using the cosine law displacement equation in Equation (15), the displacement for each interval is obtained and the cam profile is drawn using the inversion method. The default parameters of the cam mechanism of the bionic woodpecker percussion mechanism are shown in Table 3.

(13) $$H=\sqrt{x^{2}+y^{2}}$$

(14) $$h=H-r_{0}$$

(15) $$S=h[1-\cos(\pi \partial /\partial_{0})]/2$$

## 3. Experiment and Results

### 3.1. Experimental Design

#### 3.1.1. Experimental Materials and Equipment

**Figure 8:**
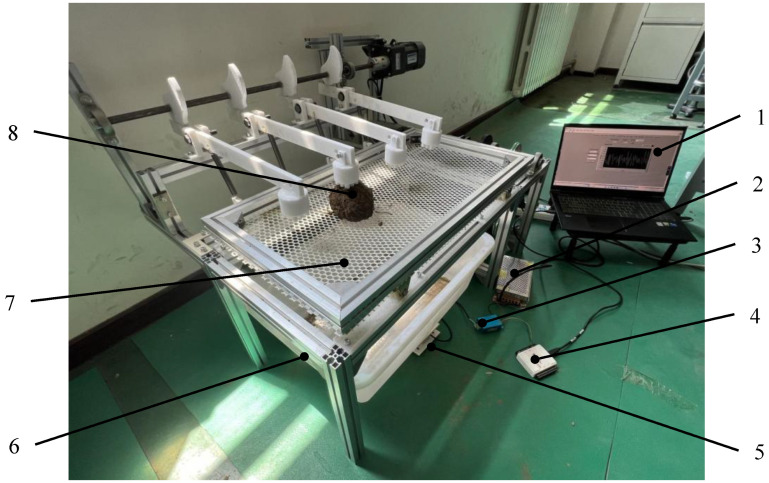
Experimental bench for the soil removal device for the root-soil complex of *Gentian* imitating the percussion of woodpeckers. 1—computer; 2—power supply; 3—signal converter; 4—data acquisition card; 5—weighing sensor; 6—soil-weighing tray; 7—experimental bench; 8—the root-soil complex of *Gentian*.

The experimental site was located at the Engineering College of Shenyang Agricultural University and the experiment materials were taken from the *Gentian* planting base in Qingyuan County, Fushun City, Liaoning Province. In order to adapt to the operational requirements of the soil removal device and reduce the experiment errors, before the experiment, the aboveground parts of the *Gentian* were removed and the root-soil complexes of *Gentian* were divided into appropriate sizes. The soil moisture content in the root-soil complexes of *Gentian* was controlled to stay between 18% and 21%, making it close to the state of the soil just out of the ground in line with the actual production situations. The experiment equipment used is a self-developed root-soil complex of a *Gentian* soil removal device imitating woodpecker percussion. The experiment prototype is shown in Figure 8. The root-soil complex of *Gentian* detached soil quality is monitored in real-time by the self-developed LabVIEW-based weight detection system; the cam speed is controlled by the speed governor on the speed motor.

#### 3.1.2. Experimental Factors

Based on the analysis combined with the structure and operating parameters of the bionic woodpecker percussion soil removal device, the three key factors were selected for the experiment to influence root-soil separation as follows.

The mass of the striking hammer *X*_1_. The striking hammer is the key unit of the bionic woodpecker-knocking soil removal mechanism and the weight of the striking hammer determines the amount of soil-breaking energy during the operation. When the mass of the striking hammer is too large, it may damage the roots of *Gentian*. According to the preliminary experiments, the range of suitable masses for the striking hammer was determined to be between 0.6 kg and 1.0 kg.

The swing angle of the striking arm *X*_2_. If the swing angle of the striking arm is too high, it will affect the energy generated when the striking hammer comes into contact with the root-soil complex of *Gentian*, even if it will lead to a higher removal rate, and also affect the damage of the roots. According to the calculations, the range of suitable swing angles for the striking arm was determined to be between 40° and 50°.

The rotation speed of the cam *X*_3_. If the rotation speed of the cam is too slow, it will lead to low efficiency of the knocking soil removal mechanism and will not be able to achieve the working effect of replacing manual labor. If the rotation speed of the cam is too high, the percussion soil removal mechanism will repeatedly knock the root-soil complex of *Gentian*, which will damage the roots of *Gentian* after soil removal. According to the preliminary experiments, the range of suitable rotation speeds for the cam was determined to be between 60 r/min and 120 r/min.

#### 3.1.3. Determination of Experimental Indicators

Based on the percussion analysis and the kinematic analysis of the bionic percussion soil removal mechanism, the mass of the striking hammer, the swing angle of the striking arm, and the rotation speed of the cam were determined as the experimental factors, while the soil removal rate and the energy consumption of a single hammer percussion were taken as the experimental indexes.

The soil removal rate (*Y*_1_) is defined as the percentage of soil removed by the device compared to the total soil mass encapsulated by the root system before being put into the device, and is calculated as follows:

(16) $$Y_{1}=\frac{W_{1}-W_{2}}{W_{1}-W_{3}}\times 100\%$$

where, *Y*_1_—soil removal rate, %; *W*_1_—mass of root-soil complex before experiment, kg; *W*_2_—mass of root-soil complex after the experiment, kg; *W*_3_—mass of the root system without soil, kg.

The energy consumption of the single hammer percussion (*Y*_2_) is defined as the energy consumption of single hammer percussion of the soil removal mechanism during the period when the bionic percussion soil removal mechanism continues to percussion the root-soil complex of *Gentian* until no further soil detachment occurs.

(17) $$Y_{1}=\frac{tX_{3}E_{p}}{60}$$

where, *Y*_2_—energy consumption of the single hammer percussion, J; *t*—time required to hammer the root-soil complex of *Gentian* until no further soil detachment occurs, s; *E_P_*—the energy consumption of the single hammer percussion, J.

### 3.2. Experimental Program and Results

In order to analyze the effects of the different striking hammer masses, the swing angle of the striking arm, and the rotation speed of the cam on the performance of the bionic knocking soil removal mechanism and to find the best combination of parameter, according to the Box–Benhken experiment theory, the ternary quadratic orthogonal regression combination experiment was designed, and the experiment factors and levels are shown in Table 4.

**Figure 9:**
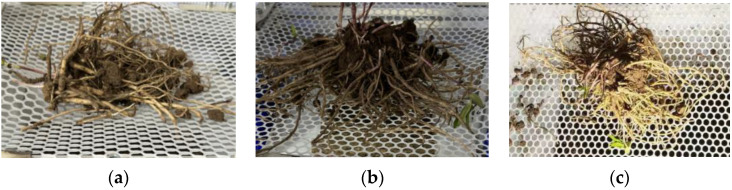
Comparison diagrams of soil removal experiment results. (**a**) is the soil removal efficiency figure under the parameter setting of No. 15 in Table 5; (**b**) is the soil removal efficiency figure under the parameter setting of No. 1 in Table 5; (**c**) is the soil removal efficiency figure under the parameter setting of No. 4 in Table 5.

The experimental scheme and results are shown in Table 5, with a total of 17 groups of experiments, including 12 analytical factors and 5 zero-point estimation errors; groups 1–12 are analytical factorization design experiments and groups 13–17 are central design experiments. Five samples were taken from each group of experiments and the average value was taken as the experiment value. A comparison of soil removal test results is shown in Figure 9.

## 4. Analysis and Discussions

### 4.1. Regression Modeling and Significant Analysis

An analysis of variance (ANOVA) was performed using Design-Expert to establish mathematical models for the regression of the soil removal rate and the energy consumption of the single hammer percussion, respectively, as a result of the experiment.

(1) Regression analysis and significance experiment of the soil removal rate

The results of the ANOVA for the soil removal rate *Y*_1_ are shown in Table 6, with a highly significant model fit (*p* < 0.0001) and a lack of fit value of 0.7406 > 0.05, indicating that the lack of fit is not significant and the fitting accuracy is high. This suggests that the model is able to predict the soil removal rate relatively accurately. The effects of *X*_1_, *X*_2_, *X*_3_, *X*_1_^2^, *X*_2_^2^, and *X*_3_^2^ on the model are highly significant and the effects of *X*_1_*X*_2_, *X*_1_*X*_3_ have significant effects on the model. The factors affecting the soil removal rate in the order of predominance are the mass of the striking hammer, the swing angle of the striking arm, and the rotation speed of the cam. Excluding the term that does not have a significant effect, the regression model for the soil removal rate *Y*_1_ is:

(18) $$\begin{array}{l} Y_{1}=89.86+2.32x_{1}+1.11x_{2}+0.5725x_{3}-0.8125x_{1}x_{2}-0.8825x_{1}x_{3}\\ -1.63x_{1}^{2}-1.41x_{2}^{2}-1.67x_{3}^{2} \end{array}$$

(2) Regression analysis and significance experiment of the energy consumption of single hammer percussion

The results of the ANOVA of the energy consumption of the single hammer percussion are shown in Table 7. The model fit is highly significant (*p* < 0.0001) and the lack of fit value of 0.8796 > 0.05 indicates that the lack of fit is not significant and the fitting accuracy is high. This suggests that the model is able to predict the energy consumption of the single hammer percussion with a relatively high accuracy. *X*_1_, *X*_2_, *X*_3_, *X*_1_*X*_2_, *X*_1_*X*_3_, *X*_1_^2^, and *X*_2_^2^ have highly significant effects on the model. The factors affecting the energy consumption of the single hammer percussion in order of predominance are the mass of the striking hammer, the rotation speed of the cam, and the swing angle of the striking arm. Excluding the term that does not have a significant effect, the regression model for the energy consumption of single hammer percussion *Y*_2_ is:

(19) $$\begin{array}{l} Y_{1}=31.83-1.49x_{1}-0.8979x_{2}-1.39x_{3}+1.11x_{1}x_{2}-1.65x_{1}x_{3}\\ +1.86x_{1}^{2}+3.12x_{2}^{2} \end{array}$$

### 4.2. Effects of Interaction Factors on Response Functions

#### 4.2.1. Effects of the Interaction Factors on the Soil Removal Rate

According to the regression model analysis, the response surfaces are plotted for the interaction terms that significantly influence the soil removal rate.

**Figure 10:**
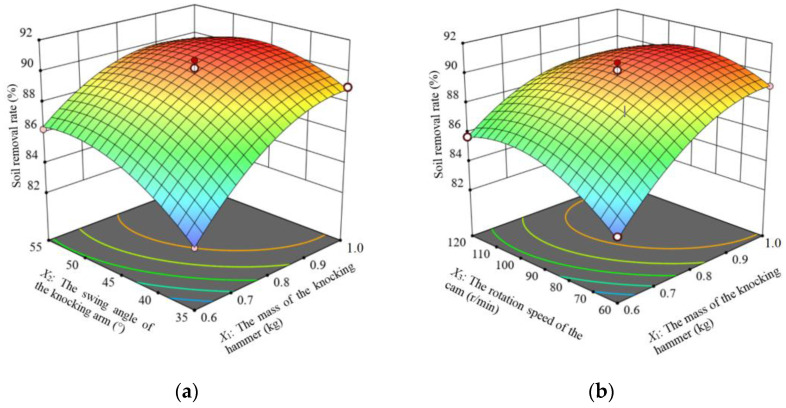
Response surface for the soil removal rate. (**a**) is *X*_3_ = 90 r/min; (**b**) is *X*_2_ = 45°.

When the rotation speed of the cam is located at the center level (90 r/min), the response surface of the interaction between the mass of the striking hammer and the swing angle of the striking arm on the effect of the soil removal rate *Y*_1_ is shown in Figure 10a. When the swing angle of the striking arm is certain, the soil removal rate with the increase in the mass of the striking hammer shows a trend of increasing slowly and then decreasing slowly. When the mass of the striking hammer is certain, the soil removal rate with the increase in the swing angle of the striking arm shows a trend of increasing rapidly and then decreasing slowly. The mass of the striking hammer and the swing angle of the striking arm interaction had an effect on the soil removal rate. In the presence of only the interaction of the two, it was found that the highest soil removal rate was achieved when the mass of the striking hammer was in the range of 0.8 to 0.9 kg and the swing angle of the striking arm was in the range of 44° to 48°.

The response surface for the effect of the mass of the striking hammer and the rotation speed of the cam interaction on the soil removal rate *Y*_1_, when the swing angle of the striking arm is located at the center level (45°), is shown in Figure 10b. When the mass of the striking hammer is certain, the soil removal rate with the increase in the rotation speed of the cam shows a trend of increasing slowly and then decreasing slowly; when the rotation speed of the cam is certain, the soil removal rate with the increase in the mass of the striking hammer shows a trend of increasing rapidly and then decreasing slowly. The interaction of the mass of the striking hammer and the rotation speed of the cam had an effect on the soil removal rate. In the presence of only the interaction of the two, it was found that the maximum soil removal rate was achieved when the mass of the striking hammer was in the range of 0.8 to 0.9 kg and the rotation speed of the cam was in the range of 90 to 110 r/min.

#### 4.2.2. Effects of Interaction Terms on Energy Consumption of the Energy Consumption of Single Hammer Percussion

According to the regression model analysis, the response surfaces are plotted for the interaction terms with significant effects on the energy consumption of the single hammer percussion.

**Figure 11:**
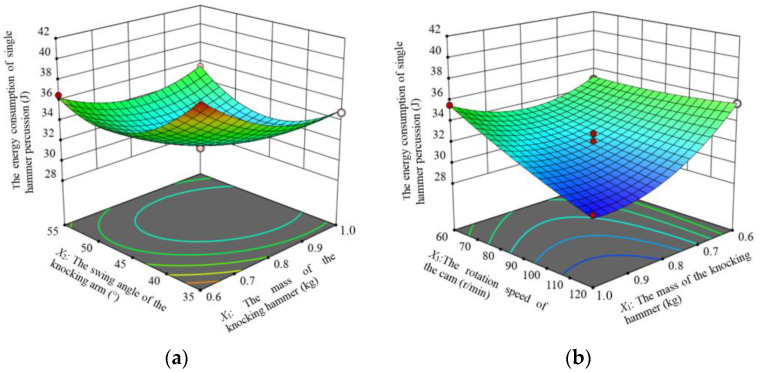
Response surface for energy consumption of a single hammer head strike. (**a**) is *X*_3_ = 90 r/min; (**b**) is *X*_2_ = 45°.

When the rotation speed of the cam is located at the center level (90 r/min), the response surface of the effect of the interaction between the mass of the striking hammer and the swing angle of the striking arm on the effect of the energy consumption of the single hammer percussion is shown in Figure 11a. When the mass of the striking hammer is certain, the energy consumption of the energy consumption of the single hammer percussion decreases and then increases with the increase in the swing angle of the striking arm. When the swing angle of the striking arm is certain, the energy consumption of the single hammer percussion increases and then decreases with the increase in the mass of the striking hammer. The mass of the striking hammer and the swing angle of the striking arm interacted to affect the energy consumption of the single hammer percussion. In the presence of only the interaction of the two, it was found that the energy consumption of the single hammer percussion was minimized when the mass of the striking hammer was in the range of 0.8–1.0 kg and the swing angle of the striking arm was in the range of 40°–50°.

When the swing angle of the striking arm is located at the center level (45°), the response surface of the interaction between the mass of the striking hammer and the rotation speed of the cam on the energy consumption of the single hammer percussion is shown in Figure 11b. When the mass of the striking hammer is certain, the energy consumption of the single hammer percussion decreases and then increases with the increase in the cam speed. When the rotation speed of the cam is certain, the energy consumption of single hammer percussion decreases and then increases with the increase in the mass of the striking hammer. The interaction of the mass of the striking hammer and the rotation speed of the cam had an effect on the energy consumption of the single hammer percussion. In the presence of only the interaction of the two, it was found that the energy consumption of the single hammer percussion was minimized when the mass of the striking hammer was in the range of 0.8 to 1.0 kg and the cam speed was in the range of 100 to 120 r/min.

### 4.3. Parameter Combination Optimization and Validation

In order to ensure that the soil removal device for the root-soil complex of *Gentian* imitates the percussion of woodpeckers with better working performance, this paper optimizes the structure and working parameters of the soil removal device according to the objectives of maximizing the soil removal rate and minimizing the energy consumption of the single hammer percussion. The optimization is solved using the Optimization-Numerical module in Design-Expert, with the objective functions and the constraint conditions being:

(20) $$\left\{\begin{array}{c} \begin{array}{c} \max Y_{1}\\ \min Y_{2} \end{array}\\ s.t.\left\{\begin{array}{c} 0.6\leq X_{1}\leq 1.0\\ 35\leq X_{2}\leq 55\\ 60\leq X_{3}\leq 120 \end{array}\right. \end{array}\right.$$

After optimization, the optimal parameter combination of the influencing factors was obtained: the mass of the striking hammer was 0.9 kg, the swing angle of the striking arm was 46.55°, and the rotation speed of the cam was 105.66 r/min; under these conditions, the soil removal rate was 90.33% and the energy consumption of single hammer percussion was 30.47 J.

The verification experiments were carried out according to the optimized parameter combination and the verification experiment parameters were as follows: the mass of the striking hammer was 0.9 kg, the swing angle of the striking arm was 47°, and the rotation speed of the cam was 100 r/min. Five replicated validation experiments under this parameter combination resulted in a mean value of 89.12% for the soil removal rate and a mean value of 31.21 J for the energy consumption of the single hammer percussion. The relative error between the experiment value of the soil removal rate and the predicted value from the regression model is 1.34% and the relative error of the energy consumption of the single hammer percussion is 2.43%, which are generally consistent with the predictions from the regression model, indicating that the optimal parameter combination is reliable.

## 5. Conclusions

(1) A soil removal device for the root-soil complex of Gentian imitating the behavior of woodpeckers striking tree trunks was designed to improve the soil removal rate and reduce power consumption.
(2) A “lever-spring-cam” striking mechanism similar to the percussion motion of woodpeckers was designed based on the analysis of the percussion motion of woodpeckers. According to the physical parameters of woodpeckers, the appropriate length of the bionic striking arm was determined. The theoretical analysis and calculation determined the range of the mass of the striking hammer, the swing angle of the striking arm, and the rotation speed of the cam.
(3) The analysis and the tests on the prototype proved that the soil removal device for the root-soil complex of Gentian imitating the percussion of woodpeckers could improve the soil removal rate and reduce the energy consumption of the single hammer percussion. Through parameter combination optimization experiments, variance analysis, and response surface analysis, the effects of factors were studied. The optimal operating parameter combination was determined as follows: the mass of the striking hammer of 0.9 kg, the swing angle of the striking arm of 47°, and the rotation speed of the cam of 100 r/min. The results of the validation experiment showed that the soil removal rate reached 89.12% and the energy consumption of the single hammer percussion was 31.21 J, which was consistent with the prediction results of the regression model and met the operational requirements of the soil removal device for the root-soil complex of *Gentian*.

## Figures and Tables

**Table 1:** The main technical parameters of the soil removal device for the root-soil complex of *Gentian* imitating the percussion of woodpeckers.

Parameters	Values/Formal
Working method	Bionic percussive
Auxiliary power/kW	0.6
Matching power speed/(r·min^−1^)	300
Dimensions (L × W × H)/(mm × mm × mm)	650 × 565 × 480
Overall quality of the machine/kg	15.0
Speed range of camshaft/(r·min^−1^)	60–120
Quality range of striking hammer/kg	0.6–1.0
Swing angle range of striking arm/(°)	35–55
Working efficiency of soil removal device/(kg·h^−1^)	40–50

**Table 2:** Geometrical parameters of the bionic woodpecker percussion mechanism.

Parameters	Values	Parameters	Values
The length of striking arm *l_AC_*/mm	488	The mass of the striking arm *m*_1_/kg	0.3
The length of swing lever *l_OC_*/mm	135	The mass of the striking hammer *m*_2_/kg	0.6 to 1.0
*l_OA_*:*l_OC_*	2.6:1	The mass of the roller *m*_3_/kg	0.1
Distance from the center of mass of the striking arm to the center of rotation *l_OB_*/mm	109	The rotation speed of the cam *n*_0_/(r/min)	60 to 120
Coefficient of spring elasticity *k*/(N/mm)	0.1	The swing angle of the striking arm *θ*/(°)	35 to 55
Distance from spring to center of rotation *l_OD_*/mm	60	*ω*_2_/(r/min)	44 to 80

**Table 3:** Default parameters table of the cam mechanism of the bionic woodpecker percussion mechanism.

Cam Rotation Angle of Push Transition Segment *φ_α_*_1_/(°)	Cam Rotation Angle of Push Segment *φ_α_*_2_/(°)	Cam Rotation Angle of Distant Rest Segment *φ_α_*_3_/(°)	The Initial Swing Angle of the Striking Arm *Ψ*_0_/(°)	The Swing Angle of Push Segment *Ψ_m_*_1_/(°)	Centre Distance *l_OE_*/mm	Base Circle Radius *r*_0_/mm	Roller Radius *r_g_*/mm
40	75	5	13	27	210	60	15

**Table 4:** Coding table for experiment factors.

Level	The Mass of the Striking Hammer *X*_1_/kg	The Swing Angle of the Striking Arm *X*_2_/°	The Rotation Speed of the Cam *X*_3_/r/min
−1	0.6	35	60
0	0.8	45	90
1	1.0	55	120

**Table 5:** Experimental design and results.

Experiment Number	Factors			*Y*_1_	*Y*_2_
	$X_{1}$	*X*_2_	*X*_3_		
1	0.6	35	90	82.56	40.3532
2	1.0	35	90	89.01	34.8355
3	0.6	55	90	86.26	36.5526
4	1.0	55	90	89.46	35.4932
5	0.6	45	60	82.97	34.9398
6	1.0	45	60	89.17	35.5581
7	0.6	45	120	85.72	35.7339
8	1.0	45	120	88.39	29.7395
9	0.8	35	60	85.44	37.6093
10	0.8	55	60	86.83	35.9547
11	0.8	35	120	85.79	34.9341
12	0.8	55	120	89.09	32.5484
13	0.8	45	90	90.23	31.3066
14	0.8	45	90	89.56	32.8719
15	0.8	45	90	90.73	31.5675
16	0.8	45	90	89.82	32.0893
17	0.8	45	90	88.96	31.3066

**Table 6:** Analysis of variance (ANOVA) for the soil removal rate.

Source	Square Sum	Degrees of Freedom	Mean Square	F-Value	*p*-Value	Significance
Model	96.80	9	10.76	38.43	<0.0001	**
*X*_1_	42.87	1	42.87	153.19	<0.0001	**
*X*_2_	9.77	1	9.77	34.90	0.0006	**
*X*_3_	2.62	1	2.62	9.37	0.0183	*
*X*_1_ *X*_2_	2.64	1	2.64	9.44	0.0180	*
*X*_1_ *X*_3_	3.12	1	3.12	11.13	0.0125	*
*X*_2_ *X*_3_	0.9120	1	0.9120	3.26	0.1140	
*X*_1_^2^	11.20	1	11.20	40.03	0.0004	**
*X*_2_^2^	8.33	1	8.33	29.75	0.0010	**
*X*_3_^2^	11.69	1	11.69	41.77	0.0003	**
Residual	1.96	7	0.2799			
Lost Proposal	0.1637	3	0.0546	0.1216	0.9426	
Pure Error	1.80	4	0.4489			
Aggregate	98.76	16				

Note: ** indicates highly significant (*p* < 0.01), * indicates significant (0.01 < *p* < 0.05).

**Table 7:** Analysis of variance (ANOVA) for the energy consumption of single hammer percussion.

Source	Square Sum	Degrees of Freedom	Mean Square	F-Value	*p*-Value	Significance
Model	115.55	9	12.84	41.03	<0.0001	**
*X*_1_	17.86	1	17.86	57.08	0.0001	**
*X*_2_	6.45	1	6.45	20.61	0.0027	**
*X*_3_	15.42	1	15.42	49.28	0.0002	**
*X*_1_ *X*_2_	4.97	1	4.97	15.88	0.0053	**
*X*_1_ *X*_3_	10.93	1	10.93	34.94	0.0006	**
*X*_2_ *X*_3_	0.1336	1	0.1336	0.4271	0.5343	
*X*_1_^2^	14.50	1	14.50	46.34	0.0003	**
*X*_2_^2^	41.11	1	41.11	131.38	< 0.0001	**
*X*_3_^2^	0.4013	1	0.4013	1.28	0.2947	
Residual	2.19	7	0.3129			
Lost Proposal	0.4206	3	0.1402	0.3169	0.8139	
Pure Error	1.77	4	0.4424			
Aggregate	117.74	16				

Note: ** indicates highly significant (*p* < 0.01).

## Data Availability

The datasets analyzed in the current study are available from the lead author on reasonable request.
